# Supplementary figures and images for: Discriminant validity of 3D joint kinematics and centre of mass displacement measured by inertial sensor technology during the unipodal stance task
Source: PLoS One. 2020 May 14;15(5):e0232513. doi: 10.1371/journal.pone.0232513 (PMC7224481; doi:10.1371/journal.pone.0232513)

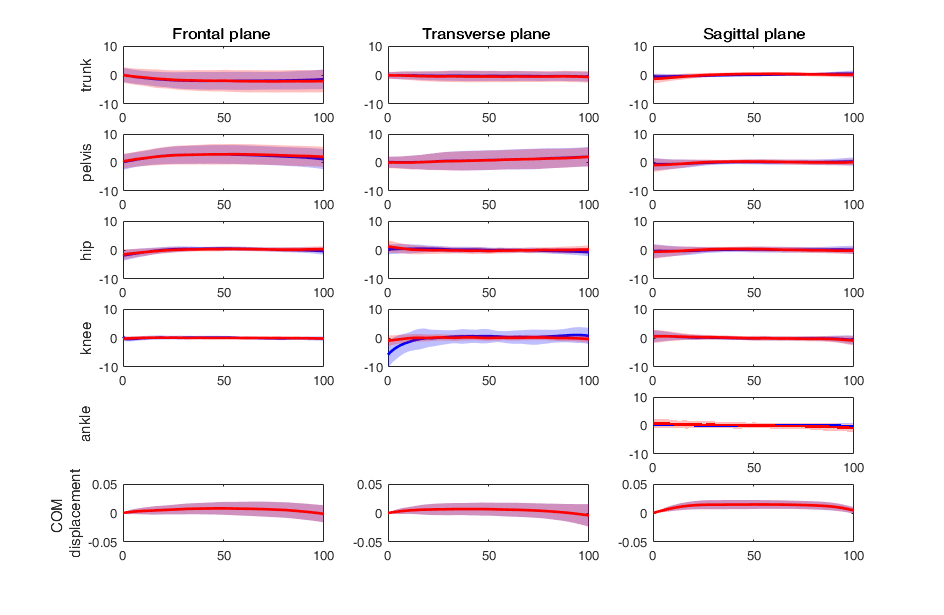

Supplement: S1 Fig — Waveform comparison between MVN BIOMECH model (red) and musculoskeletal model (blue), from pooled data healthy controls and persons with severe KOA. (TIF) [file pone.0232513.s001.tif]
