# Supplementary material for: Discriminant validity of 3D joint kinematics and centre of mass displacement measured by inertial sensor technology during the unipodal stance task
Source: PLoS One. 2020 May 14;15(5):e0232513. doi: 10.1371/journal.pone.0232513 (PMC7224481; doi:10.1371/journal.pone.0232513)
Supplement: S1 Table — (DOCX) [file pone.0232513.s002.docx]

Table S1: CMC and RMSE of 3D joint kinematics and COM displacement.

|  |  | **%NaN** | **CMC** | **RMSE (°)** |
| --- | --- | --- | --- | --- |
| Frontal | Trunk | 22 | 0,75 (± 0,24) | 1,2 (± 1,1) |
|  | Pelvis | 15 | 0,81 (± 0,21) | 1,0 (± 1,0) |
|  | Hip | 1 | 0,87 (± 0,15) | 0,5 (± 0,3) |
|  | Knee | 51 | 0,55 (± 0,2) | 0,7 (± 0,4) |
| Transverse | Trunk | 27 | 0,76 (± 0,2) | 0,9 (± 0,7) |
|  | Pelvis | 7 | 0,88 (± 0,18) | 0,7 (± 0,6) |
|  | Hip | 16 | 0,75 (± 0,19) | 1,1 (± 0,6) |
|  | Knee | 42 | 0,45 (± 0,21) | 3,1 (± 1,5) |
| Sagittal | Trunk | 16 | 0,77 (± 0,2) | 0,6 (± 0,5) |
|  | Pelvis | 4 | 0,88 (± 0,15) | 0,5 (± 0,4) |
|  | Hip | 4 | 0,83 (± 0,18) | 0,7 (± 0,5) |
|  | Knee | 7 | 0,81 (± 0,2) | 0,5 (± 0,4) |
|  | Ankle | 56 | 0,72 (± 0,24) | 1,2 (± 0,7) |
|  |  | **%NaN** | **CMC** | **RMSE (m)** |
| COM | AP | 49 | 0,63 (± 0,26) | 0,009 (± 0,006) |
|  | ML | 24 | 0,72 (± 0,21) | 0,008 (± 0,007) |
|  | Vertical | 7 | 0,84 (± 0,15) | 0,003 (± 0,002) |
