# Supplementary material for: Discriminant validity of 3D joint kinematics and centre of mass displacement measured by inertial sensor technology during the unipodal stance task
Source: PLoS One. 2020 May 14;15(5):e0232513. doi: 10.1371/journal.pone.0232513 (PMC7224481; doi:10.1371/journal.pone.0232513)
Supplement: S2 Table — (DOCX) [file pone.0232513.s003.docx]

Table S2: Reliability and agreement of 3D joint kinematics and COM displacement.

|  |  | **Within-session** | | | | | | | **Between-session** | | | | | | | **Between-operator** | | | | | | |
| --- | --- | --- | --- | --- | --- | --- | --- | --- | --- | --- | --- | --- | --- | --- | --- | --- | --- | --- | --- | --- | --- | --- |
|  |  | **ICC** | **CI** | **mean (± SD)** | **SEM** | **MDC** | **%SEM** | **%MDC** | **ICC** | **CI** | **mean (± SD)** | **SEM** | **MDC** | **%SEM** | **%MDC** | **ICC** | **CI** | **mean (± SD)** | **SEM** | **MDC** | **%SEM** | **%MDC** |
| Frontal | Trunk | 0,69 | 0,49 - 0,86 | 3,6 ± 1,9 | 1,04 | 2,88 | 28,9 | 80,0 | 0,71 | 0,25 - 0,89 | 3,5 ± 1,5 | 1,03 | 2,85 | 29,7 | 82,3 | 0,68 | 0,17 - 0,88 | 3,56 ± 1,37 | 0,93 | 2,58 | 26,2 | 72,6 |
|  | Pelvis | 0,80 | 0,63 - 0,91 | 4,4 ± 2,4 | 1,02 | 2,83 | 23,4 | 64,8 | 0,76 | 0,37 - 0,91 | 4,0 ± 2,0 | 1,13 | 3,13 | 28,3 | 78,5 | 0,70 | 0,20 - 0,89 | 4,3 ± 1,9 | 1,28 | 3,54 | 29,7 | 82,2 |
|  | Hip | 0,69 | 0,49 - 0,85 | 4,0 ± 1,9 | 1,01 | 2,79 | 25,0 | 69,3 | 0,67 | 0,20 - 0,87 | 3,9 ± 1,6 | 1,14 | 3,15 | 29,4 | 81,6 | 0,67 | 0,19 - 0,87 | 3,9 ± 1,5 | 1,07 | 2,97 | 27,3 | 75,8 |
|  | Knee | 0,42 | 0,19 - 0,68 | 1,2 ± 0,5 | 0,36 | 0,99 | 30,9 | 85,6 | 0,62 | 0,04 - 0,85 | 1,2 ± 0,4 | 0,30 | 0,85 | 25,7 | 71,2 | 0,38 | 0,00 - 0,76 | 1,2 ± 0,4 | 0,32 | 0,90 | 27,2 | 75,4 |
|  | Ankle | 0,39 | 0,16 - 0,65 | 6,8 ± 3,8 | 3,01 | 8,35 | 44,1 | 122,1 | 0,87 | 0,66 - 0,95 | 6,7 ± 2,9 | 1,46 | 4,06 | 21,7 | 60,2 | 0,83 | 0,57 - 0,93 | 7,0 ± 2,9 | 1,59 | 4,41 | 22,6 | 62,5 |
| Transverse | Trunk | 0,36 | 0,11 - 0,64 | 2,1 ± 0,8 | 0,62 | 1,71 | 29,9 | 83,0 | 0,74 | 0,32 - 0,90 | 2,1 ± 0,7 | 0,45 | 1,26 | 21,6 | 59,8 | 0,50 | 0,00 - 0,80 | 2,3 ± 0,8 | 0,57 | 1,58 | 24,3 | 67,4 |
|  | Pelvis | 0,63 | 0,41 - 0,82 | 4,1 ± 2,1 | 1,31 | 3,62 | 31,8 | 88,0 | 0,58 | 0,00 - 0,84 | 4,2 ± 1,7 | 1,37 | 3,78 | 32,9 | 91,2 | 0,27 | 0,00 - 0,74 | 4,1 ± 1,5 | 1,38 | 3,82 | 33,4 | 92,6 |
|  | Hip | 0,60 | 0,38 - 0,80 | 4,5 ± 1,7 | 1,11 | 3,08 | 24,9 | 68,9 | 0,79 | 0,46 - 0,92 | 4,6 ± 1,5 | 0,91 | 2,53 | 19,9 | 55,0 | 0,87 | 0,67 - 0,95 | 4,4 ± 1,4 | 0,68 | 1,88 | 15,3 | 42,3 |
|  | Knee | 0,49 | 0,26 - 0,73 | 4,5 ± 1,8 | 1,26 | 3,50 | 27,8 | 77,0 | 0,78 | 0,43 - 0,91 | 4,6 ± 1,5 | 0,90 | 2,49 | 19,4 | 53,9 | 0,79 | 0,48 - 0,92 | 4,5 ± 1,3 | 0,77 | 2,12 | 17,2 | 47,6 |
|  | Ankle | 0,53 | 0,30 - 0,75 | 5,6 ± 2,8 | 1,93 | 5,35 | 34,5 | 95,7 | 0,80 | 0,51 - 0,92 | 5,8 ± 2,4 | 1,39 | 3,85 | 23,9 | 66,2 | 0,91 | 0,76 - 0,96 | 5,6 ± 2,3 | 0,96 | 2,67 | 17,4 | 48,1 |
| Sagittal | Trunk | 0,66 | 0,44 - 0,84 | 3,1 ± 1,7 | 1,02 | 2,82 | 32,5 | 90,2 | 0,50 | 0,00 - 0,81 | 3,3 ± 2,1 | 1,71 | 4,74 | 51,6 | 143,0 | 0,76 | 0,34 - 0,91 | 3,2 ± 1,5 | 0,98 | 2,71 | 30,3 | 84,1 |
|  | Pelvis | 0,70 | 0,49 - 0,86 | 2,8 ± 1,5 | 0,84 | 2,34 | 30,0 | 83,2 | 0,31 | 0,00 - 0,73 | 3,2 ± 1,9 | 1,71 | 4,74 | 53,7 | 148,7 | 0,61 | 0,00 - 0,86 | 3,0 ± 1,2 | 0,90 | 2,51 | 30,5 | 84,5 |
|  | Hip | 0,53 | 0,30 - 0,75 | 3,2 ± 1,4 | 0,97 | 2,68 | 30,1 | 83,3 | 0,29 | 0,00 - 0,72 | 3,5 ± 1,8 | 1,66 | 4,59 | 46,8 | 129,6 | 0,71 | 0,24 - 0,88 | 3,3 ± 1,2 | 0,79 | 2,20 | 24,3 | 67,5 |
|  | Knee | 0,76 | 0,59 - 0,89 | 3,5 ± 1,7 | 0,79 | 2,20 | 22,5 | 62,4 | 0,75 | 0,35 - 0,90 | 3,5 ± 1,6 | 1,02 | 2,84 | 29,3 | 81,3 | 0,89 | 0,73 - 0,96 | 3,6 ± 1,6 | 0,73 | 2,03 | 20,6 | 57,1 |
|  | Ankle | 0,52 | 0,29 - 0,75 | 4,8 ± 1,9 | 1,36 | 3,76 | 28,3 | 78,5 | 0,68 | 0,17 - 0,87 | 5,4 ± 2,1 | 1,30 | 3,61 | 24,1 | 66,8 | 0,77 | 0,39 - 0,91 | 5,2 ± 1,8 | 0,98 | 2,70 | 18,7 | 51,9 |
| COM | AP | 0,43 | 0,19 - 0,68 | 0.021 ± 0,010 | 0,008 | 0,022 | 37,2 | 103,2 | 0,74 | 0,36 - 0,9 | 0.021 ± 0,007 | 0,004 | 0,012 | 21,6 | 60,0 | 0,30 | 0,00 - 0,73 | 0.021 ± 0,007 | 0,006 | 0,018 | 31,1 | 86,2 |
|  | ML | 0,43 | 0,19 - 0,68 | 0.021 ± 0,010 | 0,007 | 0,018 | 30,5 | 84,6 | 0,81 | 0,51 - 0,92 | 0.021 ± 0,009 | 0,005 | 0,014 | 18,4 | 51,1 | 0,60 | 0,00 - 0,84 | 0.027 ± 0,008 | 0,006 | 0,017 | 22,1 | 61,4 |
|  | Vertical | 0,43 | 0,19 - 0,68 | 0.021 ± 0,008 | 0,003 | 0,009 | 14,7 | 40,8 | 0,61 | 0,06 - 0,84 | 0.021 ± 0,007 | 0,005 | 0,014 | 21,8 | 60,6 | 0,86 | 0,62 - 0,95 | 0.024 ± 0,007 | 0,003 | 0,009 | 13,4 | 37,1 |
