# Supplementary material for: Discriminant validity of 3D joint kinematics and centre of mass displacement measured by inertial sensor technology during the unipodal stance task
Source: PLoS One. 2020 May 14;15(5):e0232513. doi: 10.1371/journal.pone.0232513 (PMC7224481; doi:10.1371/journal.pone.0232513)
Supplement: Attachment — Submitted filename: response to reviewers - final.pdf [file pone.0232513.s004.pdf]

Comments of reviewer 1:

Dear Reviewer,

Thank you for your constructive feedback. In an attempt to maximally address these comments, we motivate the consequent changes in the manuscript in coloured typing. The revisions in the manuscript have been implemented using “Track Changes”.

*Statistical methods of validation seem solid, although it would be advisable that they were reviewed by an expert in the field.

Before we started with the analysis of the data, we consulted Prof. dr. J. Vanrenterghem who is one of the experts that developed the SPM method for biomechanical waveform analysis [1,2]. Based on his feedback the analysis of kinematic waveforms was completed, to find differences between healthy controls (HC) and persons with knee osteoarthritis (PwKOA).

[1] T.C. Pataky, M.A. Robinson, J. Vanrenterghem, Vector field statistical analysis of kinematic and force trajectories, Journal of biomechanics. 46 (14) (2013) 2394-401.

[2] T.C. Pataky, J. Vanrenterghem, M.A. Robinson, Zero- vs. one-dimensional, parametric vs. non-parametric, and confidence interval vs. hypothesis testing procedures in one-dimensional biomechanical trajectory analysis, Journal of biomechanics. 48 (7) (2015) 1277-85.

Major concerns:

* My major concern is about the methodology/implementation of the proposed test (unipodal stance task). The test had to be performed during at least three seconds, then the participant had to come back to bear the weight in both limbs, and then repeat this sequence for five times. I am familiar with a very similar test, the single/one leg balance test, in which the individual has to stand on one leg and keep balance while bearing the weight in one leg, with the arms placed in the hip, etc. I have used this test in my own research, both with people with KOA in the waiting list for TKR surgery and with healthy matched controls. Healthy controls can probably perform this 5-time sequence test. But I am not sure that every patient with severe KOA actually can. In my experience, a considerable number of individuals cannot even bear the weight for 1-2 seconds. Even less to repeat this sequence for five times without feeling tremendous pain or losing balance. In this line, you mentioned that 19 patients were recruited. Were the 19 patients recruited able to perform the test procedure? Or do you originally recruited more than 19 individuals and excluded some of them?

The authors would like to mention that only 19 PwKOA were recruited for this study and that all patients performed the test procedure as described in the manuscript. However, the authors understand the reviewers concerns regarding the execution of the unipodal stance tasks.

First of all, the feasibility of this unipodal stance task was beforehand discussed with the three orthopaedic surgeons that are co-author of this manuscript (JM, JB & JT), and all agreed that it would be feasible for persons with severe KOA to perform the unipodal stance task.
Nevertheless, all participants were carefully instructed regarding the performance of the unipodal stance task. Before task execution, the task was explained and executed by the operator that was guiding the measurements, to give the participant an indication of how to perform the task. Subsequently the task was practiced by the participant to familiarize a uniform task execution (according to the instruction given). The instructions were given by operators that were experienced with motion analysis studies, one of them was a physiotherapist with 12 years' experience and the other a human movement scientist with 7 years' experience. The number of trials that the participant practiced were not recorded, but there were inter-participant differences observed during the measurements. The authors therefore agree with the reviewer that there were participants which had problems to maintain balance. However, all participants managed to collect 5 correctly executed trials and the number of trials that were recorded ranged between 5 to 7 trials (among all 19 participants).

Additionally, we asked the PwKOA to score their pain on a numeric pain rating scale (0 = no pain; 10 = worst possible pain). Although these results were not included within the manuscript, one participant scored a 9 just after the performance of the 5 repetitions, two participants a score of 5 and the other 16 participants had a score between 0 and 2. Therefore, based on our findings we have shown that it is feasible to perform an unipodal stance task in PwKOA.

Furthermore, based on the KOOS we showed that the PwKOA had a significantly lower scores compared to the HC. However, the larger standard deviation for the PwKOA indicates that some of the participants had more associated problems to their knees than others. Based on these findings it is possible that we have some selection bias in our population and recruited "better" patients with severe KOA. As the patients decided to participant on voluntary basis, it is possible that the patients that are unable to execute the task, or with severe pain didn't want to participate.

Therefore, the following changes were implemented within the manuscript:

Discussion, page 14/15, line 383-389
When interpreting the results of this study some limitations need to be considered. For this study both healthy controls and persons with severe KOA were included to evaluate the discriminant validity of the joint kinematics measured by the inertial sensor system. It could be argued that the persons with KOA might have difficulties with the performance of the unipodal stance task, although all the included participants performed five repetitions of the unipodal stance task according to the instructions. Inherent to this type of research is a possible selection bias. It is possible that the participants that were recruited were persons which do not cover the full KOA spectrum, given the active assessment protocol.

* The single-leg test may be a suitable test to be used with KOA patients, since the fact that the individual is not able to do it for at least 5 seconds, implies limited balance / stability or high risk of falling. However, the purpose for which this test was used in this research is otherwise: establishing knee instability by means of assessing 3D joint kinematics and the COM displacement. So the fact that patients were or not (and are or not) able to complete the sequence is an important issue

The authors understand the concern of the reviewer regarding the performance of the unipodal stance task. However, we would like to emphasize that all participants executed the unipodal stance test according to our instructions.

Furthermore, the purpose of this manuscript was to validate the inertial sensor system in order to explore the potential to measure knee joint instability and postural sway by means of center of mass (COM) displacement, as is stated in the abstract, introduction and discussion.

Abstract, page 2, line 34-37
Objective: To investigate the discriminant validity of three-dimensional joint kinematics and centre of mass displacement (COM) between healthy controls (HC) and PwKOA, during unipodal stance using inertial sensors. Additionally, the reliability, agreement and construct validity are assessed to determine the reproducibility and accuracy of the discriminating parameters.

Introduction, page 4, line 94-101
Therefore, the primary aim of this study is to investigate the discriminant validity of 3D joint kinematics and the COM displacement as measured by an inertial sensor system, by assessing differences in trunk and lower limb joint angles and postural sway (i.e. COM displacement) between HC and persons with unilateral KOA during the unipodal stance. A secondary aim of this paper is to assess the within-session, between-session and between-operator reliability and agreement of the discriminant joint kinematics and COM displacement, together with their construct validity by comparing the outcomes of the inertial sensor system against these of a camera-based motion capture system (gold standard).

Discussion, page 12, line 313-315, line 320-322
The aim of this study was to investigate the discriminant validity of 3D kinematics waveforms and COM displacement measured by an inertial sensor system during the unipodal stance task between HC and PwKOA.

Discussion, page 12, line 320-322
Additionally, the reliability, agreement and construct validity of these four discriminating parameters were investigated to verify whether these parameters could be measured in a reproducible and accurate manner.

* With respect to the test methodology, maybe I have missed something, since the manuscript has lot of technical information and it is easy to get lost, but... were they allowed to rest between repetitions? Were there a min/max time established? This certainly could influence test performance.

The authors agree with the reviewer that it was not described whether the participants were able to rest or not. However, in case the participants would like to rest this was possible, as it was important to us that the tests were executed according to our prescriptions and in a similar manner. Therefore this was added to the method section:

Methods, page 5, line 144-145
All participants were able to rest in between repetitions if required.

With respect to the second question: "*Were there a min/max time established?".* The time that the participant was standing on one leg was not established, as the purpose of this study was to determine the validity of joint kinematics and the COM displacement measured by means of an inertial sensor system. Therefore the joint kinematics of the inertial sensor system were recorded simultaneously by the inertial sensor system and the camera-system (i.e. gold standard). In case the aim of this study was to assess the performance of the unipodal stance task, the authors agree with the reviewer that the time that the participant was standing on one leg is important as this may influence the test performance. However, for the current purpose the authors do not think this is required.

Some other comments:

Abstract.

- Please spell out abbreviations at first mention.
  The abbreviations in the abstract were adjusted

- The authors state one objective in the background section. Then, there are two other objectives in the objective section. Please, clarity and consistency is recommended.
  The authors would like to mention that the objective assessment in the background section of the abstract refers to an objective assessment by means of an inertial sensor system or camera-based system, to gain insights in the knee joint stability and postural sway.

  Therefore, this line was adjusted into: *"As persons with knee osteoarthritis (PwKOA) have poor postural and knee joint stability, objective assessment of the unipodal stance task might be useful."*

L59. Please, do not use the word elderly, but older adults. This word may be considered derogatory.
Elderly persons is adjusted to older adults.

L65. The authors introduce limitations of persons with KOA, that is fine. Then the authors state which are the limitations in persons after TKA. However, individuals that have undergone TKA are not being assessed in this work, but individuals with KOA.

The authors agree with the reviewer that in line 65 indeed limitations with regard to self-reported knee joint instability (i.e. sensation of buckling, shifting or giving away the knee joint) are described. However, this is not only a problem in KOA, but also after total knee replacement (TKR) this remains a problem. In order to highlight the importance of the problem, and as many persons with end-stage KOA will receive total knee replacement surgery, we added this sentence into the introduction.

According to the authors this has no impact on the fact that we included only persons with (severe) KOA, especially because these persons were awaiting for total knee replacement. Nevertheless if the reviewer thinks that it would be better to delete this sentence, we are willing to delete it.

L114. The authors mention for the first time that the criteria included "diagnosis of unilateral KOA and awaiting for a total knee replacement (TKR) surgery". Until this point of the manuscript, the authors had referred to the target population as individuals with KOA. However, if the participants were recruited from a waiting list for TKR surgery, I wonder whether it was because there was a diagnosis indicating a severe/last stage of the condition. If so, I believe the authors should clarify that the target population is not all possible patients with KOA, but patients with severe KOA alone. To do this, information concerning the clinical / radiological diagnosis of eligible participants should also be included, and amend throughout the manuscript to refer to this population.

The authors agree with the reviewer that this could be confusing. Only persons with severe KOA were recruited for the present study. The fact that they were on the waiting list for knee surgery emphasizes the fact that only severe KOA was included. According to the reviewers comment we adjusted persons with KOA into persons with severe KOA, throughout the manuscript.

Furthermore, the orthopaedic surgeons that cooperate with this study checked additionally the severity of KOA based on the radiographic images of the individual patients included within the present study. From the 19 patients that were included, 18 had a KL-grade 4 score and one participant KL grade 3. This information was included in the results section:

Results, page 9, line 231-232
Twelve healthy controls and 19 persons with severe KOA (unilateral KOA; Kellgren / Lawrence *grade 3 (n=1) – 4 (n=18))* were recruited to investigate the discriminant & construct validity (Table 2).

L120. “Both groups completed the Knee injury and Osteoarthritis Outcome Score (KOOS) to evaluate the extent of symptomatic problems at the knee related to KOA”. This information is in the same paragraph than the inclusion criteria. But, was there a threshold or score in this test used to include/exclude participants? Otherwise this information should be placed in data collection.

The authors agree with the reviewer that this is information for the part of the methods which describes the data-collection, as no threshold of the KOOS was used to in- or exclude participants. Therefore the following changes have been made:

Methods, page 5/6, line 145-147
Before each session, both groups completed the Knee injury and Osteoarthritis Outcome Score (KOOS) to evaluate the extent of symptomatic problems at the knee related to KOA.

* At this point, I have not seen a priori simple calculation, or a post-hoc sensibility analysis to determine whether the sample size was suitable for the study purpose.

As this study is part of a larger project, justification of the number of subjects is based on an overview of compartmental forces measured in participants after TKR [3], which report an average medial compartmental force of 1.61 (±0.305) body weight during gait. Assuming an increase of 1 Stdev (0.31 BW) to be clinically significant in subjects suffering from medial compartmental OA, a sample size of 14 subjects was calculated with a of 0.05 and power level of 0.80. As it was expected that some of the participants could dropout after inclusion some additional participants were recruited. Moreover, sample sizes that vary from 10 to 25 participants are common in comparable (validation) studies [4-9], therefore we are convinced that this number of participants is sufficient.

[3] B.J. Fregly, T.F. Besier, D.G. Lloyd, S.L. Delp, S.A. Banks, M.G. Pandy, et al., Grand challenge competition to predict in vivo knee loads, Journal of orthopaedic research. 30 (4) (2012) 503-13.
[4] J.H. Bergmann, R.E. Mayagoitia, I.C. Smith, A portable system for collecting anatomical joint angles during stair ascent: a comparison with an optical tracking device, Dynamic medicine : DM. 8 (2009) 3.
[5] A. Leardini, G. Lullini, S. Giannini, L. Berti, M. Ortolani, P. Caravaggi, Validation of the angular measurements of a new inertial-measurement-unit based rehabilitation system: comparison with state-of-the-art gait analysis, Journal of neuroengineering and rehabilitation. 11 (2014) 136.
[6] C. Nuesch, E. Roos, G. Pagenstert, A. Mundermann, Measuring joint kinematics of treadmill walking and running: Comparison between an inertial sensor based system and a camera-based system, Journal of biomechanics. 57 (2017) 32-8.
[7] E. Palermo, S. Rossi, F. Marini, F. Patane, P. Cappa, Experimental evaluation of accuracy and repeatability of a novel body-to-sensor calibration procedure for inertial sensor-based gait analysis, Measurement. 52 (2014) 145-55.
[8] X. Robert-Lachaine, H. Mecheri, C. Larue, A. Plamondon, Validation of inertial measurement units with an optoelectronic system for whole-body motion analysis, Medical & biological engineering & computing. 55 (4) (2017) 609-19.
[9] [11] J.T. Zhang, A.C. Novak, B. Brouwer, Q. Li, Concurrent validation of Xsens MVN measurement of lower limb joint angular kinematics, Physiological measurement. 34 (8) (2013) N63-9.

Conclusion

The authors stated three different objectives. Conclusion mention 3D kinematics but no mention to center of mass displacement has been made. Authors also stated in the objectives that “the reliability, agreement and construct validity are assessed to determine the reproducibility and accuracy of the discriminating parameters”. But no conclusion to these parameters was made. Please align conclusions with the objectives. And clarify the objectives.

Like mentioned in the introduction the aim of this study was twofold:

Introduction, page 4, line 94-101
…the primary aim of this study is to **investigate the discriminant validity** of 3D joint kinematics and the COM displacement as measured by an inertial sensor system, by assessing differences in trunk and lower limb joint angles and postural sway (i.e. COM displacement) between HC and persons with unilateral KOA during the unipodal stance. A secondary aim of this paper is to **assess the within-session, between-session and between-operator reliability and agreement** of the discriminant joint kinematics and COM displacement, together with their construct validity by comparing the outcomes of the inertial sensor system against these of a camera-based motion capture system (gold standard).

The authors agree with the comment of the reviewer that no information with respect to the second objective was included within the conclusion of this manuscript.

Therefore, the following changes were made:

Conclusion, page 16, line 412-417
Additional discriminating parameters, which are related to *postural sway,* knee joint instability or the progression of KOA such as a greater knee adduction angle and greater knee flexion angle were measured by the camera-based system. Unfortunately, these differences *did not discriminate when measured by inertial sensors, because the detected difference in ROM between healthy control and persons with KOA was smaller as the minimum detectable change or because the construct validity was not sufficient*.

Comments of reviewer 2:

Dear Reviewer,

Thank you for your constructive feedback. In an attempt to maximally address these comments, we motivate the consequent changes in the manuscript in coloured typing. The revisions in the manuscript have been implemented using “Track Changes”.

First use of KOA, PwKOA should be written out in abstract. In line 46 "greater as" should be "greater than." In line 51 "before it" should be "before they." On line 141 MVN Biomech Awinda is produced by Xsens, not Vicon Technologies. KAM is only used on line 343 and should be written out.
The comments of the reviewer were adjusted within the manuscript.

What method was used to transform the trunk and pelvic angles to account for segment coordinate frame differences?

The authors agree with the reviewer that it was not described how the trunk and pelvic angles were transformed. Therefore the following changes have been made to the document:

Methods, page 7, line 182-187
This was required as within the musculoskeletal model the trunk was defined as one rigid body, whereas the trunk was divided into four segments in the MVN BIOMECH model. Therefore, these segments were accumulated to have a similar representation of the trunk in both models. Furthermore, the MVN BIOMECH pelvic orientation was converted into Euler angles to match the pelvic angles (expressed in the global reference frame) of the musculoskeletal model.

How was waveform normality verified?

The authors agree with the reviewer that it was not stated how the normality was verified. The normality of the waveform was obtained using Statistical Parametric Mapping, which includes a function to assess normality of the waveform for the two-sample t-test (spm1d.stats.normality.ttest2) that is based on the k2 residuals, i.e. assessment of skewness and kurtosis [10,11].

[10] R.B. D'Agostino, A. Belanger, R.B. D'Agostino, A Suggestion for Using Powerful and Informative Tests of Normality, The American Statistician. 44 (4) (1990) 316-21.
[11] http://www.spm1d.org/

Therefore, the following was added to the manuscript:

Methods, page 7, line 194-197

First, the normality of the waveforms was tested, *using the normality function of SPM for a two-sample t-test, which is based on the k2 residuals that evaluates skewness and kurtosis of the normal distribution [23]. After the normality* was verified, dependent on the outcome a parametric two-sample t-test (SPM{t}, α=0.05) or a non-parametric two-sample t-test (SnPM{t}, α=0.05) was used.

23. R.B. D'Agostino, A. Belanger, R.B. D'Agostino, A Suggestion for Using Powerful and Informative Tests of Normality, The American Statistician. 44 (4) (1990) 316-21.
