# Supplementary material for: Discriminant validity of 3D joint kinematics and centre of mass displacement measured by inertial sensor technology during the unipodal stance task
Source: PLoS One. 2020 May 14;15(5):e0232513. doi: 10.1371/journal.pone.0232513 (PMC7224481; doi:10.1371/journal.pone.0232513)
Supplement: Attachment — Submitted filename: response to reviewers - highlights rev1_Final.docx [file pone.0232513.s005.docx]

Dear editor of PlosOne,

Thank you for the opportunity to make a second revision of the manuscript according the comment of reviewer 1.

During the second review process, reviewer 1 raised a point regarding relevant information which was provided in the letter-to-the-reviewers after the first revision of the manuscript, but which was not included in the manuscript. Therefore, the authors have now included the requested information in the final version of the manuscript.

In the different paragraphs underneath, the information which was originally provided as an answer on the original question/remarks of reviewer 1 (blue colour) and which is now included in the revised manuscript, is highlighted. The revisions in the manuscript have been implemented using “Track Changes”.

Original major concerns:

* My major concern is about the methodology/implementation of the proposed test (unipodal stance task). The test had to be performed during at least three seconds, then the participant had to come back to bear the weight in both limbs, and then repeat this sequence for five times. I am familiar with a very similar test, the single/one leg balance test, in which the individual has to stand on one leg and keep balance while bearing the weight in one leg, with the arms placed in the hip, etc. I have used this test in my own research, both with people with KOA in the waiting list for TKR surgery and with healthy matched controls. Healthy controls can probably perform this 5-time sequence test. But I am not sure that every patient with severe KOA actually can. In my experience, a considerable number of individuals cannot even bear the weight for 1-2 seconds. Even less to repeat this sequence for five times without feeling tremendous pain or losing balance. In this line, you mentioned that 19 patients were recruited. Were the 19 patients recruited able to perform the test procedure? Or do you originally recruited more than 19 individuals and excluded some of them?

The following information, which was formulated as an answer to the reviewer after the first revision, was added in the second revision of the manuscript:

**Page 6, line 150-158:**

First of all, the feasibility of this unipodal stance task was beforehand discussed with the three orthopaedic surgeons that are co-author of this manuscript (JM, JB & JT), and all agreed that it would be feasible for persons with severe KOA to perform the unipodal stance task.

Nevertheless, all participants were carefully instructed regarding the performance of the unipodal stance task. Before task execution, the task was explained and executed by the operator that was guiding the measurements, to give the participant an indication of how to perform the task. Subsequently the task was practiced by the participant to familiarize a uniform task execution (according to the instruction given). The instructions were given by operators that were experienced with motion analysis studies, one of them was a physiotherapist with 12 years’ experience and the other a human movement scientist with 7 years’ experience.

**Page 16, line 405-417:**

Additionally, we asked the PwKOA to score their pain on a numeric pain rating scale (0 = no pain; 10 = worst possible pain). Although these results were not included within the manuscript, one participant scored a 9 just after the performance of the 5 repetitions, two participants a score of 5 and the other 16 participants had a score between 0 and 2. Therefore, based on our findings we have shown that it is feasible to perform an unipodal stance task in PwKOA.

Furthermore, based on the KOOS we showed that the PwKOA had a significantly lower scores compared to the HC. However, the larger standard deviation for the PwKOA indicates that some of the participants had more associated problems to their knees than others. Based on these findings it is possible that we have some selection bias in our population and recruited “better” patients with severe KOA. As the patients decided to participant on voluntary basis, it is possible that the patients that are unable to execute the task, or with severe pain didn’t want to participate.

* At this point, I have not seen a priori simple calculation, or a post-hoc sensibility analysis to determine whether the sample size was suitable for the study purpose.

The following information, which was formulated as an answer to the reviewer after the first revision, was added in the second revision of the manuscript:

**Page 5, line 129-135:**

As this study is part of a larger project, justification of the number of subjects is based on an overview of compartmental forces measured in participants after TKR [15], which report an average medial compartmental force of 1.61 (±0.305) body weight during gait. Assuming an increase of 1 Stdev (0.31 BW) to be clinically significant in subjects suffering from medial compartmental OA, a sample size of 14 subjects was calculated with a of 0.05 and power level of 0.80. As it was expected that some of the participants could dropout after inclusion some additional participants were recruited.

[15]	B.J. Fregly, T.F. Besier, D.G. Lloyd, S.L. Delp, S.A. Banks, M.G. Pandy, et al., Grand challenge competition to predict in vivo knee loads, Journal of orthopaedic research. 30 (4) (2012) 503-13.
